# Study on the Influence of Calcination Temperature of Iron Vitriol on the Coloration of Ancient Chinese Traditional Iron Red Overglaze Color

**DOI:** 10.3390/ma17122800

**Published:** 2024-06-07

**Authors:** Qijiang Li, Anjian Wu, Maolin Zhang, Jinwei Li, Jianwen Cao, Haorui Li, Yimei Jiang

**Affiliations:** 1 Research Center of Ancient Ceramic, Jingdezhen Ceramic University, Jingdezhen 333001, China; liqijiang@jcu.edu.cn (Q.L.); 2120094009@stu.jcu.edu.cn (A.W.); 003152@jcu.edu.cn (J.C.); 2320092034@stu.jcu.edu.cn (H.L.); jiangyimei@jcu.edu.cn (Y.J.); 2 Jiangxi Ceramic Heritage Conservation and Imperial Kiln Research Collaborative Innovation Center, Jingdezhen 333001, China

**Keywords:** iron vitriol calcination, α-Fe_2_O_3_, raw iron red, iron red overglaze color, coloration mechanism

## Abstract

Iron red, a traditional Jingdezhen overglaze color, is primarily colored with iron oxide (Fe_2_O_3_). In traditional processes, the main ingredient for the iron red overglaze color, raw iron red, is produced by calcining iron vitriol (FeSO_4_·7H_2_O). Analysis of ancient iron red porcelain samples indicates that the coloration is unstable, ranging from bright red to dark red and occasionally to black. Addressing this, the present study, from a ceramic technology standpoint, conducts a series of calcination experiments on industrial iron vitriol at varying temperatures. Utilizing methodologies such as differential scanning calorimetry-thermogravimetry (DSC-TG), Raman spectroscopy, X-ray diffraction (XRD), scanning electron microscopy with X-ray energy dispersive spectrometry (SEM-EDS), and optical microscopy (OM), this research scientifically explores the impact of iron vitriol’s calcination temperature on the coloration of traditional Jingdezhen iron red overglaze color. The findings indicate that from room temperature to 550 °C, the dehydration of iron vitriol resulted in the formation of Fe_2_(SO_4_)_3_ and a minimal amount of α-Fe_2_O_3_, rendering the iron red overglaze color a yellowish-red shade. At 650 °C, the coexistence of Fe_2_(SO_4_)_3_ and α-Fe_2_O_3_ imparted a brick-red color to the iron red. As the temperature was elevated to 700 °C, the desulfurization of Fe_2_(SO_4_)_3_ produced α-Fe_2_O_3_, transitioning the iron red to an orange red. With further temperature increase to 750 °C, the particle size of α-Fe_2_O_3_ grew and the crystal reflectivity decreased, resulting in a purplish-red hue. Throughout this stage, the powder remained in a single α-Fe_2_O_3_ phase. Upon further heating to 800 °C, the crystallinity of α-Fe_2_O_3_ enhanced, giving the iron red overglaze color a dark red or even black appearance.

## 1. Introduction

**Figure 1:**
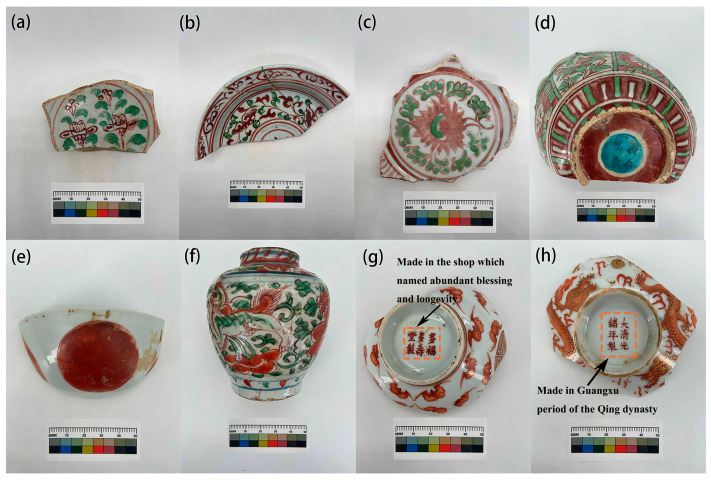
Pictures of ancient Chinese iron red from different periods. Samples (**a**,**b**) are from the Yuan Dynasty period (1271–1368), samples (**c**–**e**) are from Ming Dynasty period (1368–1644), sample (**c**) is from the mid of Ming Dynasty period, samples (**d**,**e**) are from the terminal of Ming Dynasty period, samples (**f**–**h**) are from Qing Dynasty period (1644–1911), sample (**f**) is from the mid of Qing Dynasty period, and samples (**g**,**h**) are from the terminal of Qing Dynasty period.

**Figure 2:**
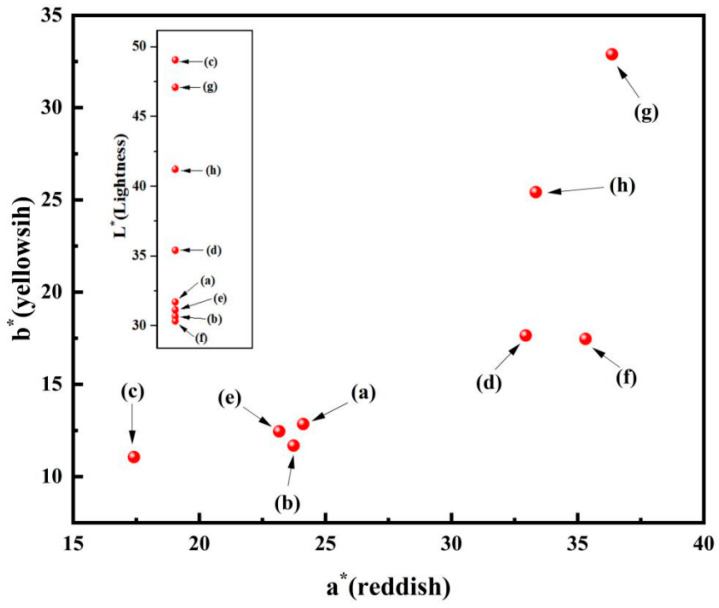
Chroma diagram of ancient samples a~h.

Iron red is a type of overglaze colorant that uses iron oxide as the pigment and can also be produced as a colored glaze when fired in an oxidizing atmosphere. Its hue often carries an orange-like red. In the mid-Ming Dynasty during the Chenghua period, the combination of underglaze blue and white with overglaze polychrome led to the production of uniquely styled doucai porcelains, which, during this period, were known for their extremely thin body and rich colors, described as “exquisite quality and vibrant colors” [1,2]. In the Jiajing period of the Ming Dynasty, the Imperial Porcelain Factory in Jingdezhen began to use iron red glaze in place of copper red glaze. By the Kangxi period of the Qing Dynasty, the color of iron red porcelain became brighter and more luxurious, typically used in the decoration of wucai (five-colors) and doucai patterns. However, after the Jiaqing period of the Qing Dynasty, the quality of the iron red overglaze color significantly declined, with only a slight improvement during the Guangxu Emperor’s reign. Therefore, the coloration of iron red overglaze color varies from period to period, and the coloration of iron red overglaze color is highly unstable (Figure 1); the chroma diagram of these samples is shown in Figure 2. 

The development of iron red has a significant historical background and plays an important role in Chinese overglaze colored porcelain. In the field of scientific archaeology, scholars have made detailed discussions on its appearance and development timeline, hues, and patterns. The production process of iron red is documented in “*Ceramic Decorative Materials*” by Wei [3], which includes drying the iron vitriol, stir-frying in an iron pot, leaching, grinding, layering, mixing with lead powder, painting, and firing to create the iron red overglaze color. “*The Science of Ancient Chinese Ceramics*” [4] mentions the production of raw iron red and proposes the view that “the color presentation of raw iron red is the final color presentation of iron red overglaze color”, a point worth exploring. Based on ancient samples of iron red ceramics, predecessors have proposed possible factors affecting coloration. During the production of iron red overglaze color, the finer the powder, the more vibrant the hue. Control over firing time and temperature must also be appropriate [5,6]. Wu et al. conducted Raman and energy dispersive X-ray fluorescence (EDXRF) analyses on iron red overglaze color and proposed factors affecting coloration such as the amount of Fe_2_O_3_, powder fineness, application thickness, and firing temperature [7]. However, there is not enough comprehensive research on the mechanistic study of its coloration. From the perspective of materials science, iron red is a red pigment synthesized from iron oxide. Some scholars have studied its phase and structure [8]. S.Kajihara et al. [9] found that at a calcination temperature of 550 °C for iron vitriol, a large number of sulfur-containing crystals was observed in the sample unit, and at 650 °C, iron vitriol was converted to α-Fe_2_O_3_ through chemical reactions. Hashimoto et al. [10] and Hirofumi et al. [11] pointed out that when the calcination temperature of raw iron vitriol reaches 700 °C, the particle size of α-Fe_2_O_3_ will increase, and the quantity decreases with rising temperature. The particle size of α-Fe_2_O_3_ is also an important factor affecting the brightness of the iron red coloration [12,13,14]. Colomban et al. [15], in his Raman study of the red colors in Qing Dynasty polychrome porcelains, indicated that the narrow characteristic peaks in the Raman spectrum show that the composition of hematite is very close to α-Fe_2_O_3_. According to the long-term practical experience of Jingdezhen potters, the calcination temperature is the largest factor affecting iron red overglaze color. The coloration of iron red overglaze color in ancient porcelains exhibits variations across different periods, while the hues presented by raw iron red at varying calcination temperatures remain unexplained from a scientific standpoint. These issues await further in-depth research.

Based on previous research, there are differences in the records of iron red pigment production in various literatures. Since many factors affect the coloration of iron red, such as the calcination temperature of iron vitriol, the fineness of the powder, the temperature for firing the painted design, and the thickness of the applied color, the most significant factor is the calcination temperature of iron vitriol. Therefore, this study focuses on investigating the impact of calcination temperature. Keeping all other experimental conditions constant, it investigates this factor using powder processed under the same conditions. The study analyzes the phase composition and changes of iron vitriol during calcination using differential scanning calorimetry-thermogravimetry (DSC-TG), Raman, and X-ray diffraction (XRD). It examines the morphology and size of the crystals with optical microscope (OM) and scanning electron microscopy-X-ray energy dispersive spectrometry (SEM-EDS) and assesses the coloration of the iron red overglaze color through chromaticity analysis. These scientific methods are employed to provide a reasonable explanation for the aforementioned historical issues.

## 2. Samples and Methods

### 2.1. Experimental Sample Processing and Results

**Figure 3:**
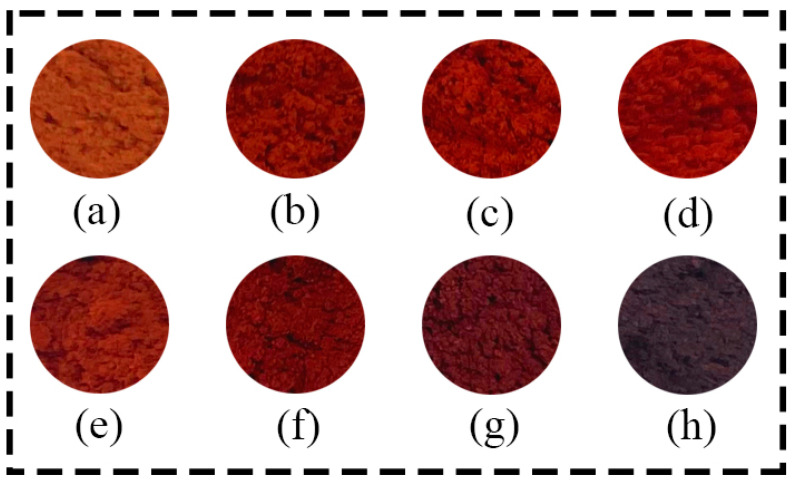
Powder images of iron vitriol calcined at different temperatures ((**a**) 550 °C; (**b**) 600 °C; (**c**) 650 °C; (**d**) 700 °C; (**e**) 750 °C; (**f**) 800 °C; (**g**) 850 °C; (**h**) 900 °C).

The raw material for the experiment was industrial iron vitriol (FeSO_4_·7H_2_O), and the supplier of industrial iron vitriol was WuHan Carnoss Co., Ltd. in Wuhan, China. This kind of industrial iron vitriol’s purity was 99%. The treatment process was as follows: Initially, the iron vitriol was preheated, with the heating temperature ranging from room temperature to 100 °C, turning its color to grayish white. Subsequently, the sample was ground to a size of approximately 250 μm. Finally, the prepared sample was placed in a muffle furnace for high-temperature calcination (Figure 3); the supplier of muffle furnace was Sigma High Temperature Electric Furnace Co., Ltd. in Luoyang, China, model SGM.M36/17A. Based on experience from multiple calcination trials, when the calcination temperature of iron vitriol reaches 550 °C, the powder turns yellow-red with a dull hue. As the calcination temperature of iron vitriol reaches 850 °C and 900 °C, the powder darkens in color. The temperature range between 550 °C and 850 °C is where red hues appear, and it was within this range that ancient potters selected the red product for crafting iron red overglaze color. Therefore, the calcination temperatures of iron vitriol were set at 550 °C, 600 °C, 650 °C, 700 °C, 750 °C, 800 °C, 850 °C, and 900 °C, the heating rate of muffle furnace was 3 °C/min, the holding time was 30 min.

**Figure 4:**
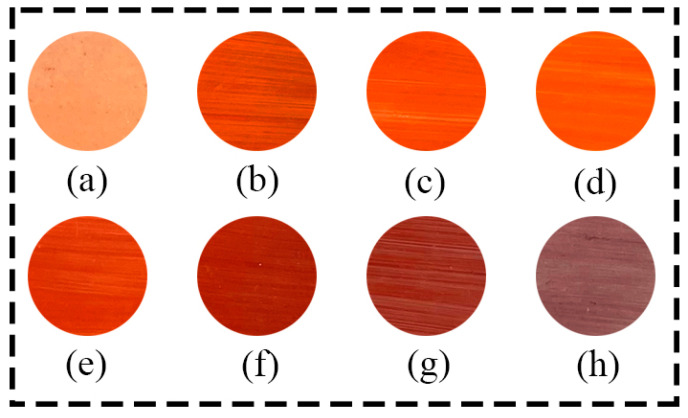
Pictures of vitriol red made of calcined iron vitriol at different temperatures ((**a**) 550 °C; (**b**) 600 °C; (**c**) 650 °C; (**d**) 700 °C; (**e**) 750 °C; (**f**) 800 °C; (**g**) 850 °C; (**h**) 900 °C).

In this experiment, following the current traditional iron red production technique in Jingdezhen, the weight ratio of raw iron red to lead pills was set at 1:4 (the weight of raw iron red was 2 g, the weight of lead pills was 8 g), and the supplier of lead pills was Jiangsu Tianxiang Chemical Group in Wuxi, China. The ball milling time for the mixture of raw iron red and lead pills was 20 min, and the color firing temperature for the iron red was 800 °C. Initially, we mixed the processed powder with 3–4 drops of frankincense oil, stirring until the two were completely integrated. Subsequently, we cut the white paper of the same thickness out the same shape and covered the white porcelain plate and applied the mixed color evenly on the white exposed plate with a hook pen. Finally, the painted iron red was placed in a muffle furnace and fired at 800 °C to produce iron red overglaze colored porcelain plates (Figure 4).

### 2.2. Test Methods

#### 2.2.1. Differential Scanning Calorimetry-Thermogravimetric Analysis (DSC-TG)

The raw material for the differential scanning calorimetric-thermogravimetric analysis was iron vitriol, and the thermal analyzer was supplied by Netzsch Instruments Manufacturing Co., Ltd. in Selb, Germany, model STA449C, with a resolution of 0.1 μg, and a temperature range from room temperature to 1650 °C. The mass of the experimental sample was 9.91 mg, with a heating rate of 10 °C/min in an Ar atmosphere. The testing temperature range was from room temperature to 900 °C, measuring the mass changes of iron vitriol and the temperature points of endothermic and exothermic reactions during heating.

#### 2.2.2. Phase Analysis

The samples for XRD testing were iron vitriol powders calcined at different temperatures. The XRD was from Bruker AXS GmbH in Karlsruhe, Germany, model D8 Advance, with a Cu target and a power of 6.5 kW; the scanning step length was 0.01° and the scanning speed was 0.05 s per step, testing the mineral composition of green vitriol during heating. Raman spectroscopy samples were powder compacts made from iron vitriol powders calcined at different temperatures using a press machine. The laser Raman spectrometer was from Renishaw in London, England, model in Via, with a spectral range of 200 nm–1000 nm and a spectral resolution of 1 cm^−1^, testing the composition of coordination compounds in iron vitriol during heating.

#### 2.2.3. Microstructure Observations and Chemical Compositions

For OM, samples consisted of thin slices made from iron vitriol powders calcined at different temperatures. The optical microscope was from Carl Zeiss in Oberkochen, Germany, model Axio Scope A1, with standard magnification ranging from 25× to 500×. It was used to examine the types and shapes of minerals formed by iron vitriol during the heating process. For SEM-EDS, samples were iron vitriol powders calcined at different temperatures. The field emission scanning electron microscope was from Hitachi in Osaka, Japan, model SU8010, with an accelerating voltage range of 0.1–30 kV, a resolution of 1 nm, and magnification ranging from 20× to 800,000×. It was used to analyze the morphology, size, and chemical composition of different crystals formed by iron vitriol during heating.

#### 2.2.4. Colorimetric Analysis

The samples for chromaticity analysis were iron red fired from iron vitriol calcined at different temperatures, and the chromaticity meter used was a NF-333 portable spectrophotometric chroma meter with a wavelength range of 400–700 nm. All measurements were made under standard 10° observation conditions and D65 illuminant to test the relevant chromaticity parameters of different iron red. L* represents the brightness of the object: 0–100, indicating from black to white; a* represents the red green of the object: a positive value indicates red, and a negative value indicates green; b* represents the yellow blue of the object: a positive value indicates yellow, and a negative value indicates blue.

## 3. Experimental Results

### 3.1. The Heating Process of Iron Vitriol

**Figure 5:**
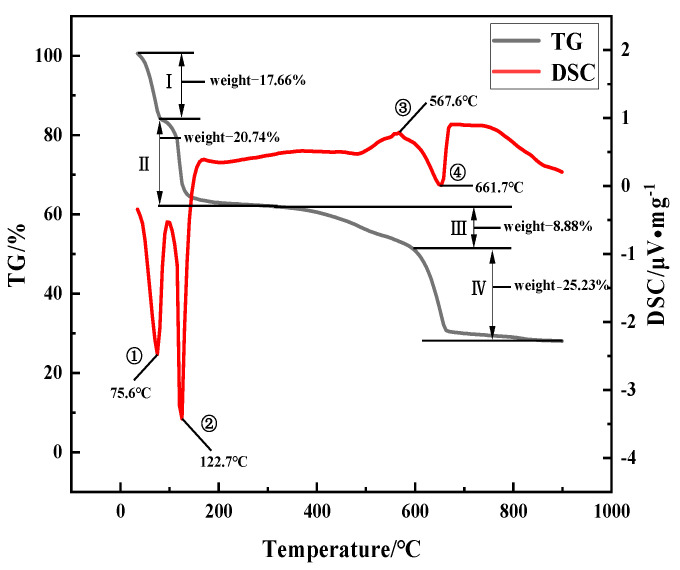
The DSC-TG curve of iron vitriol powder.

From the TG curve in Figure 5, it can be observed that there were four weight loss processes (I–IV) during the heating process, and the weight losses of the Ⅰ–IV processes were 17.66%, 20.74%, 8.88%, and 25.23%, respectively.

The DSC curve shows that three distinct endothermic reactions and one exothermic reaction occurred during the calcination process. The temperatures corresponding to ①, ②, and ④ were 75.6 °C, 122.7 °C, and 661.7 °C, respectively. The temperature for ③ was 567.6 °C.

### 3.2. Phase Variations of Iron Vitriol at Different Calcination Temperatures

#### 3.2.1. XRD

**Figure 6:**
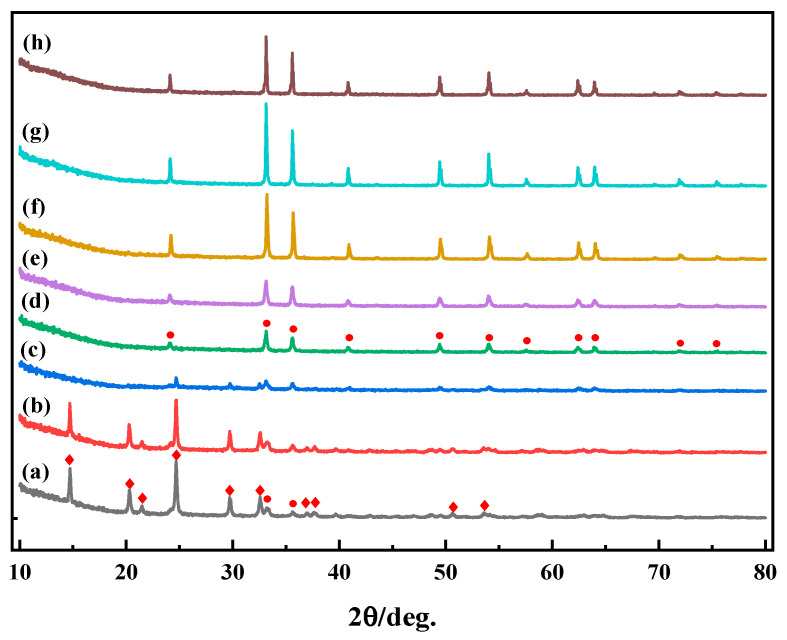
The XRD chart of iron vitriol at different calcination temperatures ((a) 550 °C; (b) 600 °C; (c) 650 °C; (d) 700 °C; (e) 750 °C; (f) 800 °C; (g) 850 °C; (h) 900 °C; • α-Fe_2_O_3_; ◆ Fe_2_(SO_4_)_3_).

From Figure 6, it can be observed that iron vitriol exhibited XRD characteristic peaks of different substances during the heating process. When the calcination temperature of iron vitriol was in the range from 550 °C to 650 °C, curves (a)–(c) showed characteristic peaks of both Fe_2_(SO_4_)_3_ and α-Fe_2_O_3_, with Fe_2_(SO_4_)_3_ being the predominant phase, and a few characteristic peaks of Fe_2_O_3_ appearing. Fe_2_(SO_4_)_3_ and α-Fe_2_O_3_ coexist during this stage [16]. When the calcination temperature of iron vitriol was in the range from 700 °C to 900 °C, in curves (d)–(h), the characteristic peaks of Fe_2_(SO_4_)_3_ disappeared, and the characteristic peaks of α-Fe_2_O_3_ became stronger. Within this temperature range, the phase was predominantly α-Fe_2_O_3_.

#### 3.2.2. Raman

**Figure 7:**
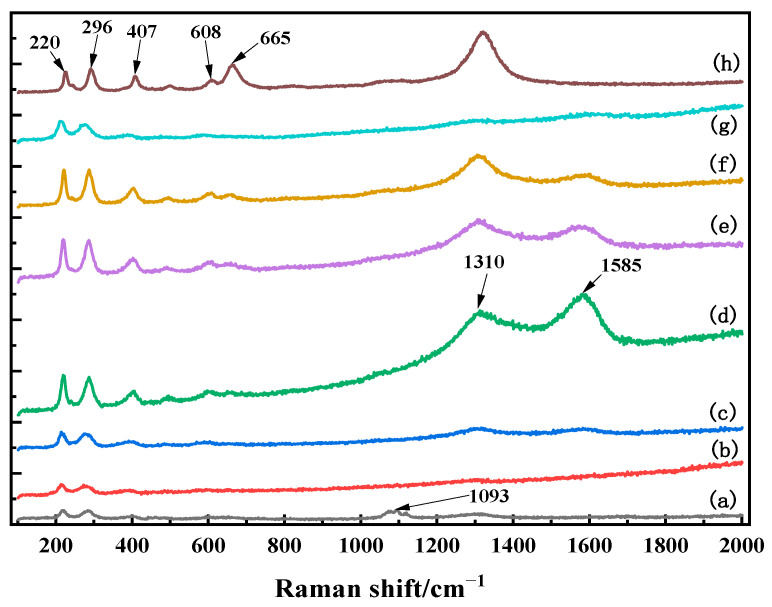
The Raman chart of iron vitriol at different calcination temperatures ((a) 550 °C; (b) 600 °C; (c) 650 °C; (d) 700 °C; (e) 750 °C; (f) 800 °C; (g) 850 °C; (h) 900 °C).

From Figure 7, it can be observed that in curves (a)–(h), the Raman spectra exhibited strong characteristic peaks at approximately 220 cm^−1^, 296 cm^−1^, 407 cm^−1^, 608 cm^−1^, 665 cm^−1^, and 1310 cm^−1^. Comparison with the Raman databases and relevant literature [17,18] indicates that these characteristic peaks correspond to the features of α-Fe_2_O_3_. Specifically, the peaks around 220 cm^−1^ and 296 cm^−1^ are typical features of α-Fe_2_O_3_ [19]. The peak around 407 cm^−1^ corresponds mainly to the symmetric bending vibration of six-coordinated Fe double-bridging oxygen, while the peak around 608 cm^−1^ corresponds to the symmetric and anti-symmetric stretching vibrations of six-coordinated Fe double-bridging oxygen [20]. The band around 1322 cm^−1^ is an overtone of the band around 669 cm^−1^, which is strongly resonantly enhanced [21]. Curve (a) shows a characteristic peak of Fe_2_(SO_4_)_3_ near 1093 cm^−1^, and there was a shift in the characteristic peak of Fe_2_(SO_4_)_3_ in this region. Due to the Raman testing process and the influence of air on the samples, all curves (a)–(h) contain a carbon phase at around 1585 cm^−1^ [17], which was not considered in the experimental results.

### 3.3. The Color of Samples

**Figure 8:**
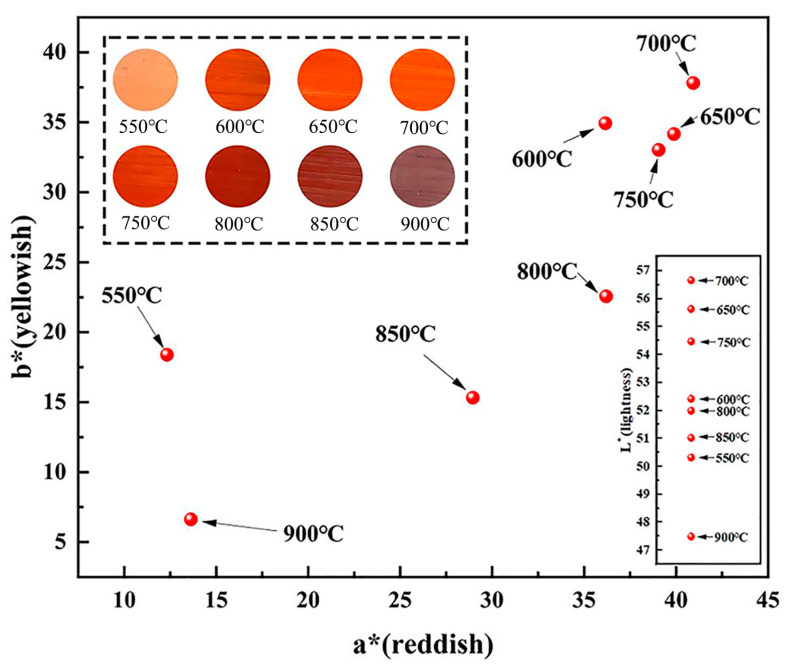
The chromaticity chart of vitriol red made of iron vitriol at different calcination temperatures.

Figure 8 shows the L*, a*, and b* values of vitriol red produced from iron vitriol at different calcination temperatures. As can be seen from Figure 6, when the calcination temperature of iron vitriol reached 550 °C to 750 °C, the a* value of vitriol red gradually increased. Conversely, when the calcination temperature was within the range from 750 °C to 900 °C, the a* value of vitriol red colors gradually decreased, with the L* value reaching its peak at 700 °C. From the results of the process experiments and chromaticity test, it can be concluded that during the heating process from 550 °C to 900 °C, the L*, a*, and b* values of iron vitriol colors all approached their maximum values when the calcination temperature was 700 °C.

### 3.4. Microstructure Analysis

**Figure 9:**
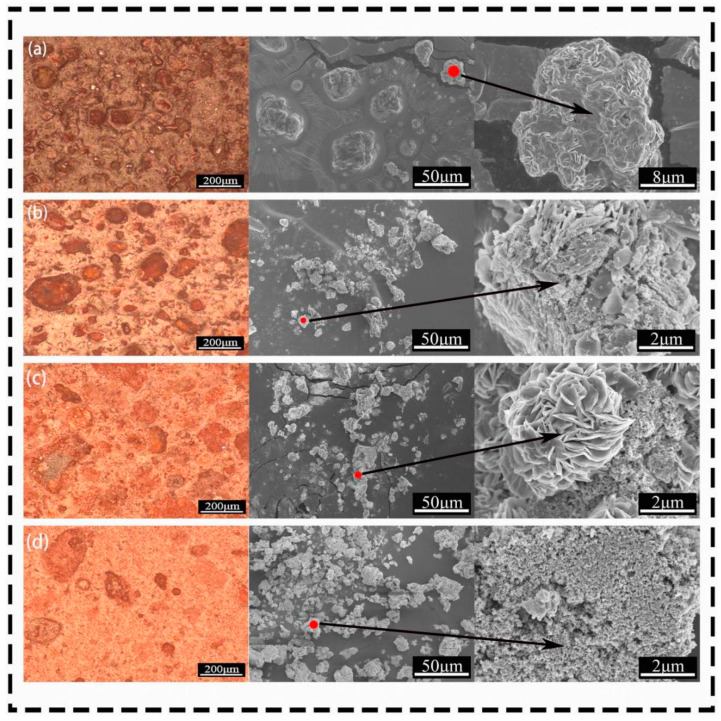

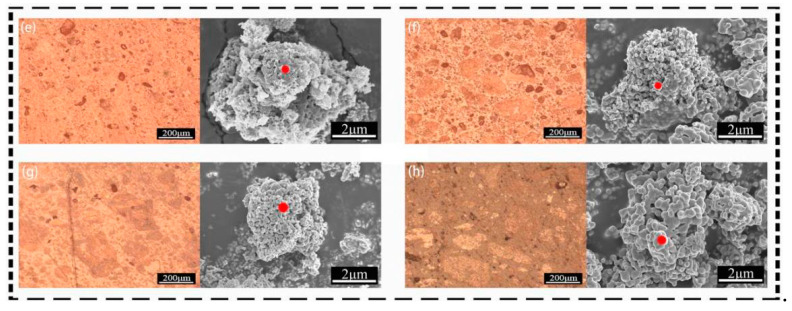
The OM and SEM images of iron vitriol at different calcination temperatures ((**a**) 550 °C; (**b**) 600 °C; (**c**) 650 °C; (**d**) 700 °C; (**e**) 750 °C; (**f**) 800 °C; (**g**) 850 °C; (**h**) 900 °C).

From the OM images in Figure 9, it can be seen that during the entire process of increasing calcination temperature, the color changed from yellow-red to orange-red to brick-red, and finally became darker. In Figure 9a–c, large blocky particles are present, while in Figure 9d–h, these large particles disappear, the crystal grains become smaller, and the overall appearance is of small particle clusters. From the SEM images, it can be observed that when the calcination temperature of iron vitriol was 550 °C, the particles were mostly blocky; we used the SmileView tool 2.1.9.9 to calculate the particle size, ranging in size from 10 to 50 μm, with a flaky layered morphology, and very few blocky particles were attached to spherical microparticles. When the calcination temperature ranged from 550 °C to 650 °C, in Figure 9a–c, numerous spherical particles could be seen growing on the surface around the blocky particles. When the calcination temperature of iron vitriol reached 700 °C, the blocky particles disappeared, and the spherical particles became larger, but their size was still relatively small at 700 °C, with an average diameter of about 70 nm. Continuing to heat to 800 °C, the particle size of α-Fe_2_O_3_ increased, with an average diameter of about 120 nm. When the calcination temperature reached 900 °C, the α-Fe_2_O_3_ particles became fuller in shape, with an average diameter of about 400 nm. According to EDS results, when the calcination temperature of iron vitriol was above 700 °C, as shown in Figure 9e–h and Table 1, the overall elemental distribution consisted of iron and oxygen, with the sulfur disappearing, and no other elements present.

## 4. Discussion

In this study, the experimental results show that the calcination temperature of iron vitriol has a relatively significant effect on the coloring of iron red overglaze color, which was proved by the following factors through experiments.

### 4.1. Phase Variations of Iron Vitriol at Different Calcination Temperatures

Through XRD and Raman results, it was found that the changes in mineral phase of iron vitriol during calcination affected the color change of the iron red overglaze color. When the calcination temperature rose from room temperature to 550 °C, both XRD and Raman results showed characteristic peaks of Fe_2_(SO_4_)_3_ and a small amount of α-Fe_2_O_3_, resulting in a yellow-red color for the iron red overglaze color. As the temperature increased to 700 °C, the XRD and Raman results showed only characteristic peaks of α-Fe_2_O_3_, indicating that Fe_2_(SO_4_)_3_ began to oxidize and formed α-Fe_2_O_3_, changing the color of the iron red overglaze color to orange-red. As the temperature continued to rise, the brightness of the raw iron red powder decreased and gradually darkened, with the powder maintaining a single-phase Fe_2_O_3_ throughout this period. According to the results of the phase analysis, the darkening of the color was not due to the formation of new substances like FeO or Fe_3_O_4_ [9]. The phase variation is the key to the initial low-temperature calcination coloration of the raw iron red, with the presence of Fe_2_(SO_4_)_3_ particles directly affecting the color of the iron red overglaze color.

### 4.2. Relationship between Sample Color and Microstructure

Through SEM-EDS and OM testing, it was found that the changes in the microstructure of iron vitriol during calcination affected the color change of the iron red overglaze color. The OM images (Figure 9) show that throughout the entire process of increasing calcination temperature, the color changed from yellow-red to brick-red to orange-red and then to date-red, eventually darkening. When the calcination temperature of iron vitriol was 550 °C, the particles were mostly blocky, corresponding to the large particles in the OM images, with sizes ranging from 10 to 50 μm and a flaky layered morphology. Combined with phase analysis, it can be inferred that these were Fe_2_(SO_4_)_3_ particles at this temperature, with very few blocky particles attached to spherical microparticles, indicating the initial formation of α-Fe_2_O_3_ at a calcination temperature of 550 °C. When the calcination temperature was between 550 °C and 650 °C, numerous spherical particles could be seen growing around the blocky particles in Figure 9a–c, indicating the coexistence of Fe_2_(SO_4_)_3_ and α-Fe_2_O_3_. As the calcination temperature of iron vitriol increased, at 700 °C, the blocky particles disappeared, and the spherical particles enlarged, with the iron vitriol powder being in a single-phase α-Fe_2_O_3_. However, the particle size at 700 °C was still relatively small, with an average diameter of about 70 nm. Factors such as the size of the α-Fe_2_O_3_ microparticles, crystal sintering degree [22], spatial distribution, and clustering affected the coloration of the iron red overglaze color. At this stage, the high reflectivity of the α-Fe_2_O_3_ surface means that low-wavelength components of the visible light spectrum are absorbed by the small-sized α-Fe_2_O_3_ microparticles, transmitting red light and resulting in a brighter red color. As the temperature further increased to 800 °C, the particle size of α-Fe_2_O_3_ increased to an average diameter of about 120 nm. The change in spatial distribution of the microparticles, influenced by the shape and size of agglomerated particles connected by individual particles, led to a decrease in reflectivity with larger agglomerated particles in space, causing the powder color to darken [12,13]. When the temperature further rose to 900 °C, the α-Fe_2_O_3_ particles continued to grow larger and fuller, with an average diameter of about 400 nm. The crystallinity of α-Fe_2_O_3_ increased, the crystal particles sintered, and the reflectivity of the crystals further decreased, resulting in the iron vitriol calcined powder turning black. From the SEM images, it can be seen that the clustering of α-Fe_2_O microparticles also led to a gradual deepening of the color of the iron red overglaze color, thereby reducing its brightness.

### 4.3. The Heating Process of Iron Vitriol

In the heating process of iron vitriol, the weight loss curve shows four distinct stages (I–IV) of weight loss. Stages I and II involve the two-step dehydration process of FeSO_4_·7H_2_O, during which the free crystalline water continuously evaporates at low temperatures, leading to the dehydration of FeSO_4_·7H_2_O to form FeSO_4_·H_2_O [23]. The main reaction in stage III is the complete dehydration of FeSO_4_·H_2_O to produce FeSO_4_, followed by the heating of FeSO_4_ to form Fe_2_(SO_4_)_3_. In this process, FeSO_4_ also reacts with O_2_, forming the intermediate product Fe_2_O(SO_4_)_2_ [24]. Stage IV begins at around 600 °C, corresponding to the decomposition of FeSO_4_ into Fe_2_(SO_4_)_3_, Fe_2_O_3_, and SO^3^. At 661 °C, the desulfurization and oxidation of Fe_2_(SO_4_)_3_ and Fe_2_O(SO_4_)_2_ led to the formation of α-Fe_2_O_3_, a compound relatively stable at high temperatures. The DSC curve indicates three distinct endothermic reactions during calcination. The first two endothermic peaks (① and ②) correspond to the process of FeSO_4_·7H_2_O losing six molecules of crystalline water, transforming into FeSO_4_·H_2_O at 250 °C. The appearance of peak ③ near 567 °C, accompanied by a slight exothermic reaction, occurred when FeSO_4_ reacted with O_2_, resulting in the intermediate product Fe_2_O(SO_4_)_2_. The peak ④ near 661 °C corresponds to the desulfurization and decomposition of Fe_2_(SO_4_)_3_ and Fe_2_O(SO_4_)_2_ to form α-Fe_2_O_3_. The temperature continued to rise to 900 °C without further chemical reactions. Therefore, the following chemical reactions occurred during the heating process of iron vitriol:

(1) $${\mathrm{FeSO}}_{4}\cdot {7H}_{2}O\;\overset{heating}{\to }{\mathrm{FeSO}}_{4}\cdot H_{2}O$$

(2) $${\mathrm{FeSO}}_{4}\cdot H_{2}O\overset{200\;{}^{\circ}C–300\;{}^{\circ}C}{\to }{\mathrm{FeSO}}_{4}{+H}_{2}O$$

(3) $${4\mathrm{FeSO}}_{4}{+O}_{2}\overset{567\;{}^{\circ}C}{\to }{2\mathrm{Fe}}_{2}O{\left({\mathrm{SO}}_{4}\right)}_{2}$$

(4) $${6\mathrm{FeSO}}_{4}\overset{550\;{}^{\circ}C-610\;{}^{\circ}C}{\to }{\mathrm{Fe}}_{2}{\left({\mathrm{SO}}_{4}\right)}_{3}{+2\mathrm{Fe}}_{2}O_{3}{+3\mathrm{SO}}_{2}$$

(5) $${\mathrm{Fe}}_{2}{\left({\mathrm{SO}}_{4}\right)}_{3}\overset{661\;{}^{\circ}C}{\to }{\mathrm{Fe}}_{2}O_{3}{+3\mathrm{SO}}_{3}$$

(6) $${\mathrm{Fe}}_{2}O{\left({\mathrm{SO}}_{4}\right)}_{2}{\overset{661\;{}^{\circ}C}{\to }}^{\;}{\mathrm{Fe}}_{2}O_{3}{+2\mathrm{SO}}_{3}$$

The reason for the color differences in the iron red overglaze color at different stages is as follows: When the calcination temperature of iron vitriol was around 550 °C, the prevailing phase of the powder predominantly comprised Fe_2_(SO_4_)_3_. The coloration caused by the presence of Fe_2_(SO_4_)_3_ crystals led to a darker hue of the iron red overglaze color, showing a yellow-red color. When the calcination temperature reached around 650 °C, Fe_2_(SO_4_)_3_ coexisted with α-Fe_2_O_3_, and at this point, the coloration of the iron red overglaze color was influenced by the α-Fe_2_O_3_ crystals, turning the hue to brick red. Around 700 °C, Fe_2_(SO_4_)_3_ had completely desulfurized, eliminating the coloration effect of Fe_2_(SO_4_)_3_ crystals. Simultaneously, α-Fe_2_O_3_ crystals had just formed with small particle size, resulting in high reflectivity. The low-wavelength components of the visible light spectrum were absorbed by the small-sized α-Fe_2_O_3_ microparticles, thus transmitting red light and presenting an orange-red color. The increase in temperature caused the agglomerated particles between crystals to enlarge, and the increase in crystal particle size, crystal sintering, and crystallinity collectively contributed to a decrease in the brightness of the powder color. Consequently, at temperatures ranging from 750 °C to 900 °C, the corresponding colors were date red, dark red, and black-red, respectively.

## 5. Conclusions

The coloration mechanism of iron red overglaze color, as derived from the preparation of raw iron red and various analysis method, such as DSC-TG, RS, XRD, SEM-EDS, and OM, yielded the following conclusions:

(1) The phase composition is the key to the coloration of raw iron red during the thermal calcination (550–900 °C), with α-Fe_2_O_3_ particles being the main colorants. The presence of Fe_2_(SO_4_)_3_ crystals directly affects the coloration of the iron red overglaze color.
(2) The size of α-Fe_2_O_3_ particles plays a crucial role in determining the brightness of the iron red overglaze color.
(3) The variation in calcination temperature of raw iron red emerges as a significant factor contributing to the diverse colorations of iron red overglaze color in various periods of ancient China.

## Figures and Tables

**Table 1:** EDS elemental atomic masses of iron vitriol samples at different calcination temperatures (Atomic%).

	O	Fe	S
550 °C	76.363	9.026	14.611
600 °C	52.304	24.444	23.251
650 °C	67.687	14.098	18.215
700 °C	63.952	32.409	3.639
750 °C	62.758	37.242	-
800 °C	64.368	35.632	-
850 °C	56.280	43.720	-
900 °C	54.855	45.145	-

Note: At the red dots in Figure 9.

## Data Availability

Data are contained within the article.
